# Preventing c2c12 muscular cells damage combining magnesium and potassium with vitamin D3 and curcumin

**DOI:** 10.1016/j.jtcme.2021.05.003

**Published:** 2021-06-02

**Authors:** Claudio Molinari, Sara Ruga, Mahitab Farghali, Rebecca Galla, Ahmad Bassiouny, Francesca Uberti

**Affiliations:** a Laboratory of Physiology, Department of Translational Medicine, University of Piemonte Orientale, via Solaroli 17, 28100, Novara, Italy; b Department of Biochemistry, Faculty of Science, Alexandria University, Alexandria, Egypt

**Keywords:** C2C12 cells, Fatigue, Muscle recovery, Nutraceuticals, Physical activity

## Abstract

**Background and aim:**

Physical activity is defined as any bodily movement produced by skeletal muscles which causes energy consumption; moderate and constant physical activity is known to be beneficial and to slow the muscle loss process associated with aging. The aim of the present study was to test, in an *in vitro* exercise model, the biological effects of a new formulation composed of magnesium and potassium combined with vitamin D and curcumin created to support muscle activity and to prevent hypercontraction damage.

**Experimental procedure:**

C2C12 cells were treated with vitamin D, buffered magnesium bisglycinate, curcumin, and potassium citrate. Cell viability, morpho-functional changes, calcium and magnesium movements, and the main kinases involved in glucose uptake were analyzed. The glycogen level and lactate were also evaluated.

**Results and conclusion:**

Important results about a positive effect on mitochondrial activity, ATP production, oxygen consumption and in the physiological differentiation of C2C12 cells were obtained. Further experiments were performed under conditions that mimic the biological aspects of strenuous exercise. The combination of magnesium, vitamin D3, curcumin, and potassium citrate revealed beneficial effects on skeletal muscle cells under physiological conditions as well as while mimicking intense activity. In particular, in an *in vitro* model, they were able to control the hypercontraction, restoring ion fluxes, reducing inflammation signaling and supporting the main mechanism involved on aerobic activity. Our results have indicated for the first time that this new combination could be considered as a new nutraceutical formulation to improve physical performance and muscle recovery.

## Highlights of the findings and novelties

• MKVC is able to counteract the negative effect of strenuous muscle activity.
• MKVC is able to restore muscle functions also in post-trauma condition.
• MKVC may have numerous roles during muscle activity and recovery.
• MKVC prevents a negative condition of strong state contraction.

## Graphical abstract

**Figure:**
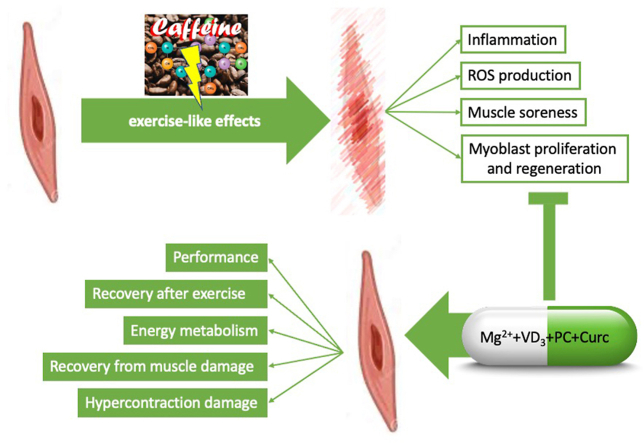

## Abbreviations

VD: vitamin D
M: magnesium buffer bysglicinate chelate
K: potassium citrate
C: curcumin
MKCVD: magnesium buffer bysglicinate chelate + potassium + citrate + curcumin + vitamin D
ROS: Reactive oxygen species
AMPK 5′: adenosine monophosphate-activated protein kinase
Glut4: Glucose transport 4
SMA: smooth muscle antibody

## 1 Introduction

Besides contraction, the skeletal muscle has several more functions, including glucose uptake because of insulin action, modulation of bone density and protein homeostasis throughout the body.^1^ Skeletal muscle mass is regulated by muscle protein synthesis processes which are controlled by reactivity to anabolic stimuli, such as food consumption or physical activity, and by protein and amino acid turnover mechanisms. Catabolic stressors such as illness, physical inactivity and inflammation tend to generate higher rates of muscle breakdown.^2^ Additionally, ROS production during strenuous exercise can contribute to the development of acute muscle fatigue.^3^ If exposure to high ROS becomes chronic, it can impair cell function leading to apoptosis and cell necrosis. In this context, dietary supplementation may be a choice to counteract the negative impact of strenuous exercise. In case of muscle injury, an inflammatory response is initiated to guide repair, and although excessive inflammation can be harmful, trying to dramatically reduce inflammation may not be the ideal solution for optimal recovery.^4^^,^^5^ For this reason, in recent decades, both elite and amateur athletes have increased the use of herbal products and dietary supplements capable of promoting muscle growth and reducing fat mass,^6^ both before and after physical activity. These are usually nutritional antioxidants that act as free radical scavengers^7^ by preventing or reducing oxidative stress, decreasing muscle pain and physical stress, and improving sports performance.^8^ Recent research has focused on the effects of nutraceutical compounds. such as curcumin, a hydroxycinnamic acid derivative present in the turmeric spice, a vegetable alkaloid obtained from the ground rhizome of the perennial plant Curcuma longa^9^ which has multiple bio-functional activities including anti-inflammatory, antioxidant, anticancer, antidiabetic and chemopreventive activities.^10^ Indeed, it has been shown that curcumin plays an important role in muscle regeneration following muscle injuries.^11^ Particularly during *in vivo* exercise experiments curcumin accelerated mitochondrial biogenesis in skeletal muscle by regulating NF-κB and PGC-1α pathways, reducing MDA level and increasing SIRT1 protein in muscle.^12^ Furthermore, in young men, prolonged ingestion of curcumin attenuates some muscle damage-related markers such as the reduction of the maximum voluntary contraction force and the expression of creatine kinase activity, supporting the hypothesis of beneficial effects after oral intake on the recovery from muscle damage induced by eccentric exercise.^13^ Strategies to promote recovery through the use of micronutrients and/or supplements include vitamin D and minerals such as magnesium and potassium. An *in vivo* study explored the role of vitamin D on muscle regeneration via satellite cell activation followed by myoblast proliferation, migration and differentiation^14^ confirming that vitamin D treatment improves cell migration and myotube differentiation, after mechanical damage,^15^ indicating a role of vitamin D in dynamic repair of skeletal muscle. Similarly, in recreationally active humans subjected to reduced exercise, the pre-exercise vitamin D status was found to be significantly correlated with immediate and long-term (48- and 72-h) muscle weakness following strenuous exercise with one leg exercised relative to the control leg.^16^ All of these findings explain the benefit of vitamin D for muscle damage repair and recovery process after exercise, including interaction with protein intake.^17^ Furthermore, the great importance of magnesium for muscle function must be taken into consideration.^18^, ^19^, ^20^ Indeed, it participates in energy metabolism and helps normal muscle contraction and relaxation.^21^ Furthermore, serum magnesium levels in male athletes^22^ or in the elderly^23^ have been shown to be associated with muscle performance, and the onset of exercise-associated muscle cramps.^21^ The results of animal studies have suggested that magnesium supplementation can improve the efficiency of energy metabolism, while human studies have indicated that it can improve performance parameters in both aerobic and anaerobic exercise.^21^ Muscle activity is also associated with an increase in extracellular potassium concentration.^24^ In dietary supplements, potassium is often present in different forms such as chloride, citrate, phosphate, aspartate, bicarbonate, and gluconate,^25^ but only a few studies have examined the effectiveness of various potassium forms in the absorption characteristics of dietary supplements. On the basis of results reported in the literature, it is possible to hypothesize several possible synergistic effects of these substances. However, to confirm the mechanistic importance of specific skeletal muscle changes in exercise-mediated health benefits, *in vitro* experiments performed on skeletal muscle cells require “exercise-like treatment” conditions. This approach allows investigations, directly on skeletal muscle cells, on the cellular effects of individual aspects of exercise physiology, such as skeletal muscle contraction or activation of signaling pathways that respond to exercise.^26^ Several compounds, including caffeine, have been used *in vitro* to study the molecular regulation of exercise-induced skeletal muscle adaptations.^26^ Caffeine is an ionophore for Ca2+ capable of triggering the release of Ca2+ from the sarcoplasmic reticulum and increasing the concentration of intracellular Ca2+ in skeletal muscle,^27^ For this reason, it has been used in studying the effects of activating the exercise-like CaMKII pathway in skeletal muscle myotubes. In particular, it has been applied to myotubes as a Ca2+ ionophore, with the specific intent of providing an “exercise-like treatment",^28^ that mimics the typical activation of exercise^29^^,^^30^ or exercise signaling^31^ and trigger Ca2+ changes similar to those in exercise.^32^ The aim of this study was to verify, on muscle cell cultures, the possible cooperative effects of a new combination composed of magnesium, potassium, vitamin D3, and curcumin, which could represent a new nutraceutical formulation to improve physical performance and promote muscle recovery after exercise, comparing them with the effects of individual agents. The combination of the four substances was also tested in an *in vitro* experimental model of exercise capable of mimicking the biological conditions typical of a strenuous contraction. In these experiments, caffeine was used, which is able to reproduce what happens in exercise *in vivo*. Furthermore, it has been studied whether this combination may be able to restore negative consequences of exercise such as inflammation and fatigue caused by strenuous activity, which *in vivo* lead to delayed-onset muscle soreness.

## 2 Methodology

### 2.1 C2C12 cell culture

C2C12 murine myoblasts were purchased from American Type Culture Collection (Manassas, VA, USA). Cells were grown in Dulbecco's Modified Eagle Medium (DMEM; Sigma-Aldrich, Milan, Italy) supplemented with 10% FBS (Sigma-Aldrich, Milan, Italy), 100U/ml penicillin/streptomycin and maintained in an incubator at 37 °C, 5% CO_2_ and maintained at 40–70% density to obtain cell growth (proliferation) without cell differentiation. Depending on the various experimental protocols used, 1 × 10^4^ cells were plated on 96-well plates to study the mitochondrial activity by MTT test and ATP level; 4 × 10^4^ cells were plated in black 96-well plates to study oxygen consumption and mitochondrial membrane potential; 10 × 10^4^ cells/well were seeded in six-well plates to investigate with Western blot the intracellular pathways activated, to analyze the activities by ELISA and the Glucose/Glycogen and lactate productions. To synchronize the cell cycle, C2C12 cells were incubated with DMEM without red phenol for 24 h before stimulation. The growth medium was changed with 2%FBS, containing various several agents alone and combined for a time ranging from 1 h to 8 h and 24 h–72 h to verify the early phase of differentiation and the main mechanism activated. This condition was maintained during all experimental protocols used. The effects of vitamin D3, magnesium bisglycinate, potassium citrate, and curcumin were tested in the presence or absence of a high concentration of caffeine (2.5 mM) an agent that causes calcium release from the SR, to mimic the effects of muscle contraction.^33^

### 2.2 Experimental protocol

C2C12 cell line was used to evaluate the effects of vitamin D3, magnesium bisglycinate, potassium citrate, and curcumin alone or in combination to support the cellular mechanisms underlying exercise. In addition, the role of severe contraction caused by caffeine as a side effect was investigated in presence of the more effective combination of the substances tested alone to prevent or restore the muscle damage. This study was divided into three phases. In the first phase, a dose-dependent study of the single agents (ranging from 1 h to 24 h) on cell viability was investigated to exclude negative effects. In particular, the concentrations of the substances were obtained from the literature: vitamin D3 (V) 0.001μM–1μM^34^; 15% magnesium bisglycinate chelate buffered (M) 0.1μM-5mM^35^; potassium citrate (K) 0.1mM–5mM^36^; curcumin (C) 1μM–100 μM were tested.^37^ The main effects observed were obtained by M and K 1 mM, VD 100 nM and C 100 μM, and these concentrations were used together to verify the properties of a new combination, named MKVC, in the second and third phases of the study. In the second phase, the main intracellular mechanisms underlying exercise were investigated and in the third phase, the same analyses were carried out in presence of caffeine 2.5 mM to mimic a hypercontractility condition.^32^ All substances were prepared directly into medium in a 10X concentration without adding other agents and directly used to obtain the reported final concentration.

### 2.3 Cell viability

Cell viability was analyzed at the end of each stimulation using MTT-based *in vitro* Toxicology Assay Kit (Sigma-Aldrich, Milan, Italy), as previously described.^38^ Details of the method are given in Supplementary 1.

### 2.4 Oxygen consumption and mitochondrial membrane potential

The oxygen consumption and mitochondrial membrane potential were immediately and simultaneously quantified by Oxygen Consumption/Mitomembrane Potential Dual Assay Kit (Cayman Chemical Company; Ann Arbor, MI, USA) following the manufacturer's instructions as reported in literature (details are given in Supplementary 2).^39^

### 2.5 ATP assay

At the end of each stimulation, the cells were immediately treated with the components of the ATP assay kit (Calbiochem, San Diego, USA), following the manufacturer's instructions (details are given in Supplementary 3).^40^

### 2.6 [Mg^2+^]i movements

Intracellular Magnesium concentration ([Mg2+]i) was measured using Mg2+-sensitive fluorescent dye Mag-fura-2AM (Furaptra, Biotium) as previously described.^38^ Briefly, the cells were incubated in a Hanks salt solution (Thermo Fisher Scientific, Waltham, USA) without Mg2+ containing 10 mM glucose and supplemented with 20 mM HEPES/Tris (pH 7.4), 1.3 mM CaCl_2_, and 5 μM Mag-fura-2AM at 37 °C for 30 min. The cells loaded fluorescent with Mag-fura-2AM was monitored at regular intervals starting from 3min to 300min (15, 30, 60, 120, 150, 180, 210, 240, 270) with excitation at 340 nm and 380 nm. The acquisitions were obtained using a Fluorescence Spectrometer VICTORX4 multilabel plate reader at an emission wavelength of 510 nm with an exposure time of 100 ms. The fluorescence ratios (340/380 nm) were calculated and compared to control; Rmax and Rmin were analyzed by the addition of 50 mM MgCl_2_ and 100 mM EDTA, respectively.^41^

### 2.7 [Ca2+] movements

Calcium movements analysis was performed following a classical technique.^38^ Cells have been washed twice with sterile PBS 1 × and incubated with 5 μM Fura-2AM (Sigma-Aldrich, Milan, Italy) for 30 min in the dark in PSS buffer without Ca2+ (1.5 mM KCl, 10 mM HEPES, 10 mM d-Glucose, 2 mM l-Glutamine, pH 7.4), under shaking at 37 °C. After the stimulations, the fluorescence was measured by a fluorescence spectrometer (VICTORX4 multilabel plate reader) at excitation wavelengths of 340 nm and 510 nm for emission. The results were reported as means ± SD% compared to control cells.

### 2.8 TNFα assay

TNF-α concentration in C2C12 cells was determined using TNFα ELISA kit (Sigma, Milan, Italy) according to the manufacturer's instructions (details are given in Supplementary 4).^42^

### 2.9 Glucose uptake

Cells were lysed in the extraction buffer as reported on the manufacturer's instruction of Glucose Uptake Colorimetric Assay Kit (Sigma-Aldrich) (details are given in Supplementary 5).^43^

### 2.10 Glycogen measurement

The glycogen synthesis assay was performed following the manufacturer's instructions (BioVision, Life Research, Scoresby Victoria, Australia). (details are given in Supplementary 6).^44^

### 2.11 Lactate measurement

As reported in literature^45^ the quantification of lactate level on C2C12 cells was measured after lyses on supernatants using a lactate assay kit (BioVision) according to the manufacturer's instructions. The contents of pyruvate and lactate were normalized to the total protein amount.

### 2.12 Akt activation assay

PI3K/Akt activities were measured by the InstantOneTM ELISA (Thermo Fisher, Milan, Italy) following the manufacturer's instructions (details are given in Supplementary 7).^46^

### 2.13 Phospho-p38/MAPK ELISA test

The phosphorylation levels of p38/MAPK were analyzed on cells lysates following the manufacturer's instructions as reported on Supplementary 8 (ELISA Kit, Abcam).^47^

### 2.14 Cell lysates and Western blot

At the end of each treatment, C2C12 cells were washed twice with ice-PBS 1X and lysed in ice using Ripa Buffer (50 mM Hepes, 150 mM NaCl, 0,1% SDS, 1%TRITON 100x, 1% deoxycholate acid, 10% glycerol, 1,5 mM MgCl2, 1 mM EGTA, 1 mM NaF; all purchased from Sigma-Aldrich) supplemented with 2 mM sodium orthovanadate (Sigma-Aldrich), 1 mM phenylmethanesulfonyl fluoride (PMSF; Sigma-Aldrich) and 1:100 mix Protease Inhibitor Cocktail (Sigma-Aldrich). 40 μg of protein of each sample was resolved into 8% and 15% SDS-PAGE gels, and polyvinylidene difluoride (PVDF) membranes (GE Healthcare) were incubated overnight at 4 °C with specific primary antibody: anti-rabbit Cyclin D1 (1:1000, Cell Signalling), anti-mouse Desmin (1:1000, Santa-Cruz), anti-rabbit Phospho-AMPK (1:1000, Millipore), anti-mouse AMPK (1:500, Santa-Cruz), anti-mouse Phospho-JNK (1:500, Santa-Cruz), anti-mouse JNK (1:500, Santa-Cruz) and anti-mouse SMA (1:1000, Santa-Cruz). Protein expression was normalized and verified through anti-mouse β-actin detection (1:5000; Sigma-Aldrich) and reported as mean ± SD (% vs control).

### 2.15 In vitro experimental model for examining the skeletal muscle cell biology of exercise

Due to its powerful ergogenic and metabolic stimulating effects in skeletal muscle, caffeine has been used in a series of experiments to mimic exercise-like conditions to test the MKVC combination in a hypercontractility scenario. Caffeine was used at 2.5 mM, as suggested by studies in the literature.^33^

### 2.16 Statistical analysis

Data reported were obtained from at least five independent experiments performed in triplicates for each experimental protocol and analyzed using Prism GraphPad statistical software. Results are reported as means ± SD using One-way ANOVA followed by Bonferroni post hoc test for statistical analysis. p values < 0.05 were considered statistically significant.

## 3 Results

### 3.1 Dose-response and time-dependent study of cell viability on C2C12 cells

**Fig. 1:**
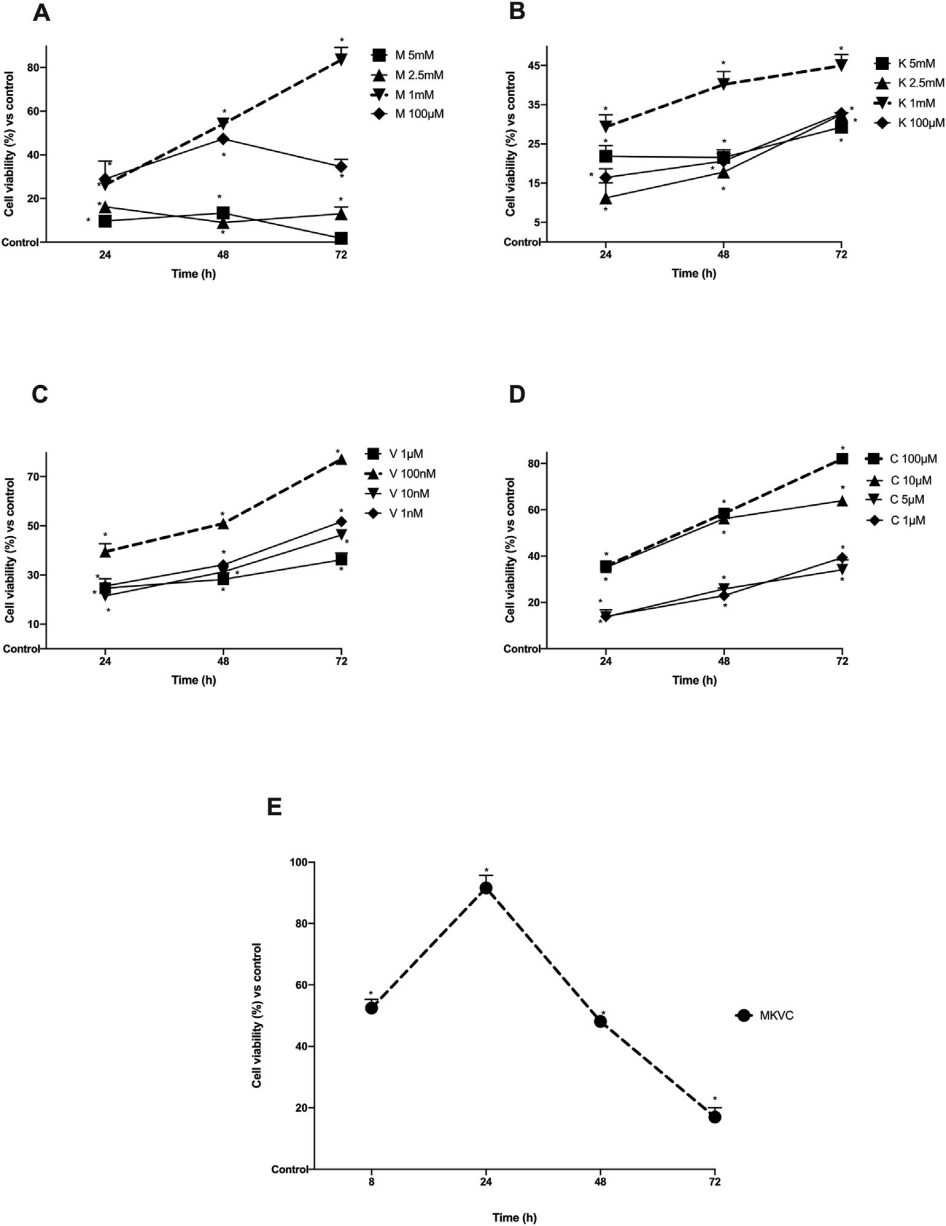
Cell viability measured by MTT. From panel **A** to **D** single agents were tested alone in a dose-response and time-dependent study. M = magnesium in panel a; K = potassium citrate in panel b; V = vitamin D3 in panel c; C = curcumin in panel d. The best dose chosen from the tested range of individual agents is indicated in red. Data reported are expressed as means ± SD of five independent experiments. ∗p < 0.05 vs control. In **E** the cell viability of the combined product named MKVC. MKVC = magnesium, potassium citrate, vitamin D and curcumin added together. ∗p < 0.05 vs control.

To verify the effects and to exclude any cytotoxic effects, C2C12 cells were stimulated with magnesium (M), potassium citrate (K), vitamin D3 (V) and curcumin (C) alone in dose-response and time-dependent experiments (24–72 h) by analyzing cell viability using MTT assay. As shown in Fig. 1A–D, in the first set of experiments all substances were able to induce an increase in cell viability during the time without cytotoxic effects. In particular, M and K (Fig. 1A and B, respectively) showed an inversely proportional effect where the 1 mM concentration was able to induce a greater effect than the other concentrations throughout the analyzed period (p < 0.05). V and C (Fig. 1C and D, respectively) showed a directly proportional effect to the dose used and V 100 nM and C 100 μM were able to induce greater effects than other concentrations tested throughout the period analyzed. All these data confirmed that none of the substances had cytotoxic effect overtime on muscle cells and 1 mM for M, 1 mM for K, 100 nM for V and 100 μM for C were the concentrations tested in combination and maintained in all subsequent experiments. As reported in Fig. 1E, the effect of the combination of tested substances, named MKVC, was also investigated on cell viability to verify its effectiveness and to exclude any negative effects. Data confirmed a time-dependent effect with a maximum effect at 24 h compared to control (p < 0.05, about 92%) and to all other time points (p < 0.05, about 52% at 8 h, 48% at 8 h and 17% at 72 h), indicating the effectiveness of the combination in stabilizing the effects of individual agents on mitochondrial health over time.

### 3.2 Morpho-functional changes in C2C12 cells treated with MKVC

**Fig. 2:**
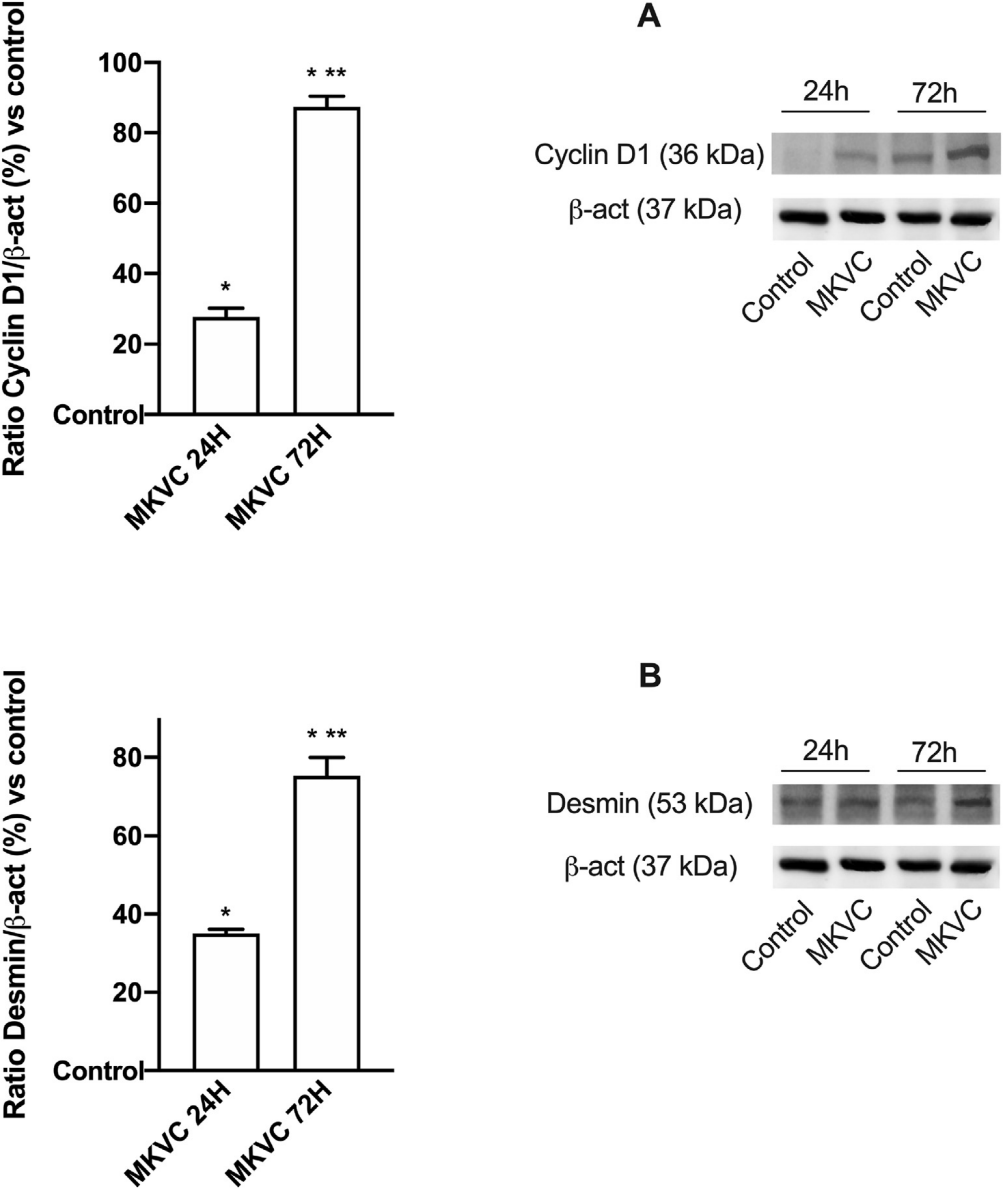
Western blot and densitometric analysis. In **A** cyclin D1 and in **B** desmin expressions caused by MKVC during time (at 24 h and 72 h). An example of Western blot normalized through β-act is reported. Data are reported as means ± SD of six independent experiments. The abbreviations are the same as reported in Fig. 1. ∗p < 0.05 vs control; ∗∗p < 0.05 vs 24 h of stimulation.

To understand if this new combination was also able to induce a physiological differentiation on myotubes of C2C12 cells, further experiments were carried out by stimulating the cells for 24 h and 72 h analyzing the differentiation phases, using cyclin D1 and desmin by Western blot analysis (Fig. 2A and B, respectively). The MKVC combination was able to induce a greater effect than the control (p < 0.05) confirming that cyclin D1 expression is abundant in proliferating myoblasts and similar data were derived from desmin analysis. Furthermore, this combination induced a greater effect at 72 h than at 24 h (p < 0.05 vs control) indicating that differentiation began at 24 h (about 27.7% and 35% on both proteins, respectively) confirming the hypothesis of better effect in conditions of intense activity.

**Fig. 3:**
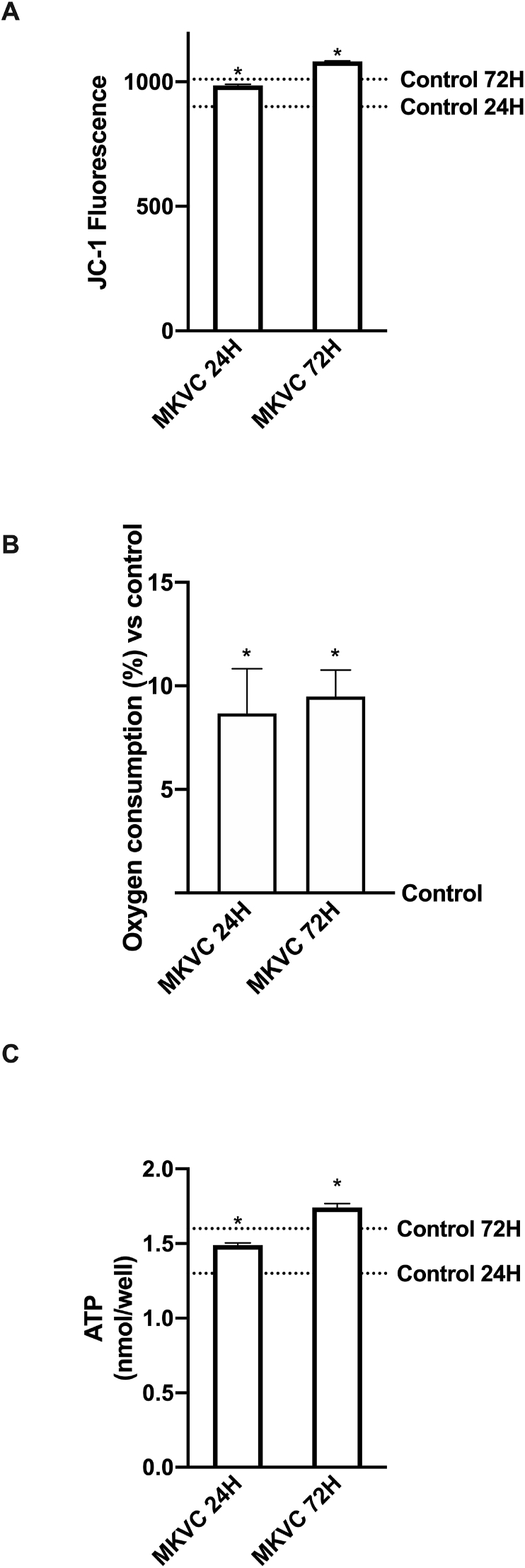
Analysis of cellular energy. In **A** mitochondrial potential membrane, in **B** oxygen consumption and in **C** ATP levels caused by MKVC during time (at 24 h and 72 h). Data are reported as means ± SD of six independent experiments. The abbreviations are the same as reported in Fig. 1. ∗p < 0.05 vs control.

Mitochondrial activity is crucial for myoblast proliferation, and it is always accompanied by increased ATP production and oxygen consumption. The analysis of mitochondria by JC1 suggested that MKVC has a significant impact on mitochondrial activity, particularly on mitochondrial membrane potential. It was able to induce a greater increase compared to control (p < 0.05) at 24 h versus 72 h (about 9% and about 7%, respectively), as shown in Fig. 3A, confirming a physiological increase in the chemical energy of the cells. In order to obtain more information on mitochondrial metabolism during exercise, oxygen consumption and ATP production were also studied. As shown in Fig. 3B and C, the rate of oxygen consumption and ATP production played an important role in myoblast proliferation. Indeed, MKVC induced an increase in oxygen and ATP consumption compared to the control (p < 0.05) and between the two times at 24 h, no significant changes were observed that could indicate the physiological activity of the mitochondria.

### 3.3 Calcium-magnesium flux analysis to determine the effects on biology of contraction-relaxation cycle

**Fig. 4:**
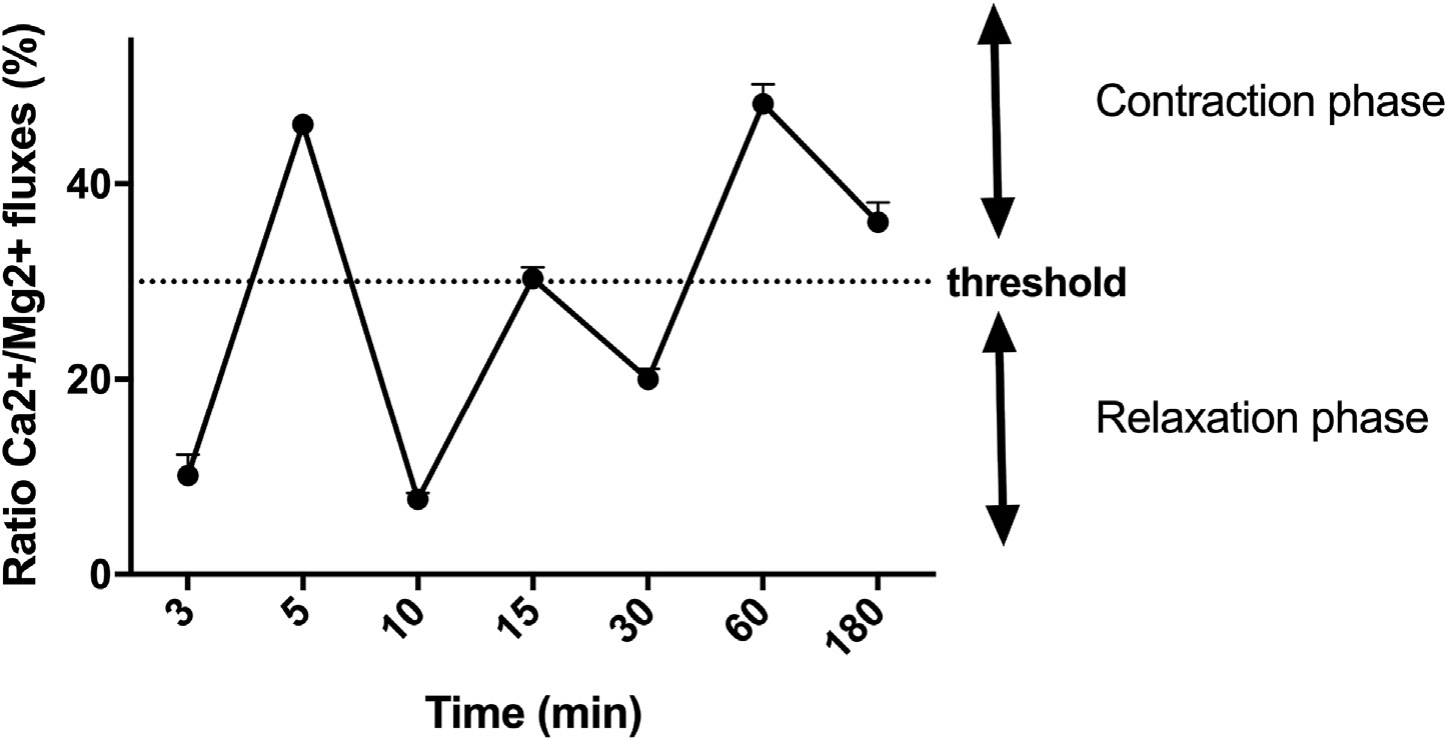
Effect on contraction of MKVC. The graph shows a ratio from calcium movements and magnesium flux normalized to control values ranging from 3min to 180min. After the administration of MKVC (time 0), a series of oscillations of the Ca2 + /Mg2 + ratio was observed, which correspond to cycles of contraction and relaxation of the cells. Data are reported as means ± SD (%) of five independent experiments. All time points are ∗p < 0.05 vs control.

As it is known, muscle contraction during exercise depends on variations in the intracellular concentrations of calcium ions (Ca2 +). Since the behavior of calcium in C2C12 cells is like that observed in *in vivo* experiments, changes in calcium and magnesium levels by Fura-2AM and Furaptra were analyzed *in vitro*, respectively. These experiments were carried out to clarify the importance of Mg2+ and Ca2+ movements after treatments with MKVC under free-Mg2+ and Ca2+ medium conditions. The balance between calcium and magnesium movements is a reference of the cycles of contraction and relaxation and, as reported in Fig. 4, it showed an alternating pattern from 3 min to 180 min with respect to the control (p > 0.05). The presence of magnesium in the formulation produces a physiological movement of calcium ions. This up and down flow was supposed to be related to the contraction-relaxation cycle. MKVC showed a significant effect over time that was significant at 5 min and 60 min (45% and 47% respectively), supporting MKVC effects on the contraction-relaxation cycle. In addition, these effects regulate a physiological balance between Mg2+ and Ca2+ flux. Data suggest that MKVC is able to reduce electrolyte flux peaks also at a physiological level, indicating lower energy consumption during muscle activity over time.

### 3.4 Assessment of muscle activity and inflammation in C2C12 cells

**Fig. 5:**
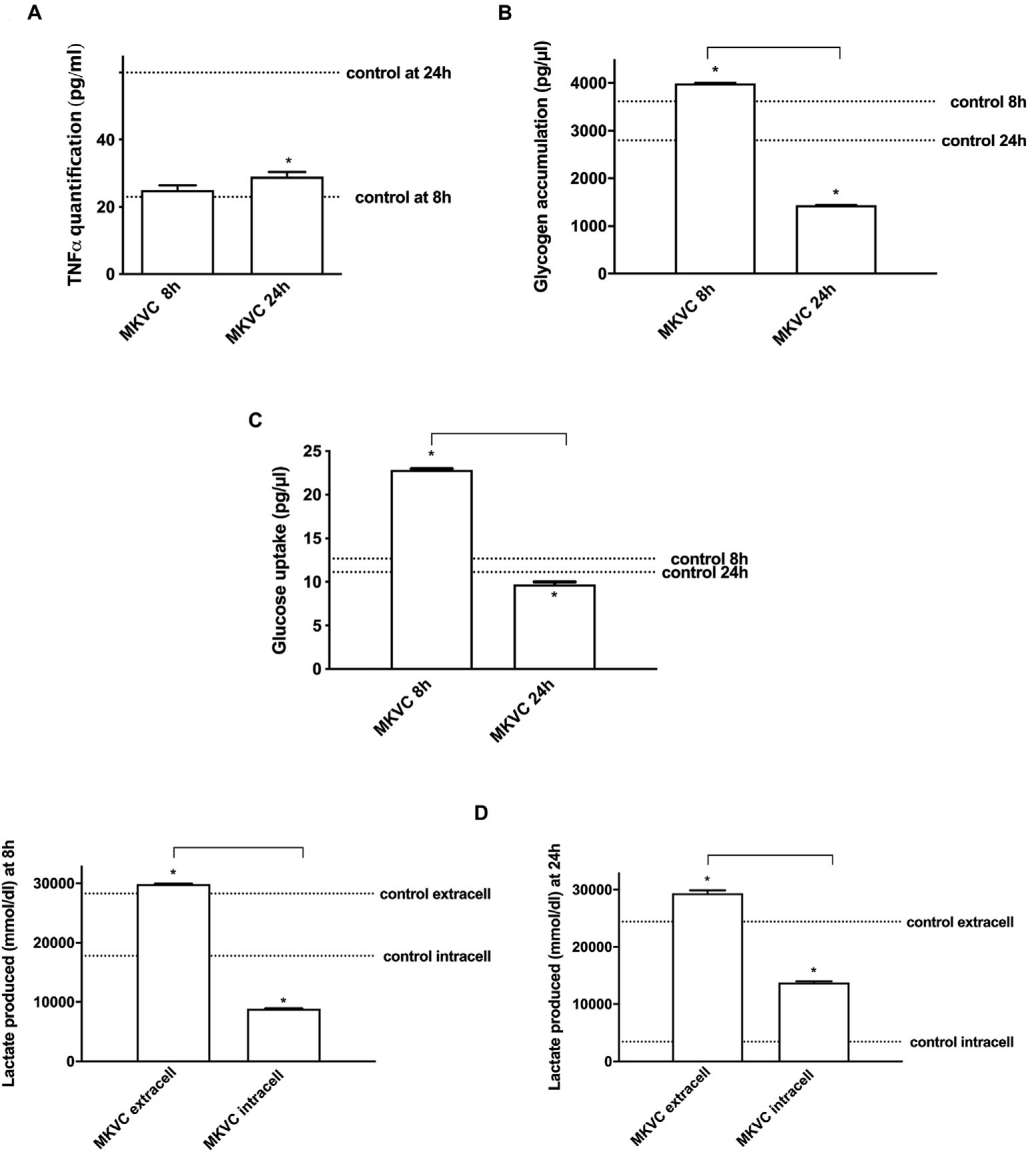
Analysis of several contraction parameters. In **A** TNF-α ELISA assay, in **B** glycogen accumulation, in **C** glucose uptake, and in **D** lactate analysis with intracellular and extracellular quantification. All these parameters were investigated at two time points. The abbreviations are the same as used in Fig. 1. Data are reported as means ± SD of five independent experiments. ∗p < 0.05 vs control; the bars p < 0.05 between the different time points.

Following the treatment, an immune response could be triggered to assist myoblast proliferation. Since TNFα expression can increase myoblast proliferation, to understand if pro-inflammatory cytokines play a role in this context, TNFα quantification was performed on C1C12 myoblast at the two-time point chosen on the effects on cell viability at 8 h (the beginning of effect) and 24 h (maximum effect). As shown in Fig. 5A, MKVC induced TNF-α released at 8 h hours and 24 h (about 8.7% respect with control and 16% respect with 8 h) in a physiological way; indeed at 8 h the production was near the control values (p > 0.05) and at 24 h the production was less than control (p < 0.05) indicating that no inflammation response was taking place. Furthermore, since MKVC administration results in normal cytokine production under physiological conditions over time, the data suggest that this formulation may be more effective in hypercontractility conditions. Since muscle is the most important user of glucose, the accumulation and degradation of glycogen in skeletal muscle plays a central role in systemic glucose homeostasis. To evaluate glucose/lactate concentration and glycogen accumulation, C2C12 cells were treated with MKVC and their respective analysis were performed. As shown in Fig. 5B and C, glycogen and glucose concentrations were more present in the myoblast at 8 h than 24 h compared to control (p < 0.05) (about 10.5% and 79% respectively). The glucose concentration showed the same trend as the glycogen variation suggesting that these two main reserves of muscle fuels were consumed under physiological conditions after MKVC stimulation. At the base of this mechanism there is also the lactate role, which is a metabolic intermediate mainly produced in muscles under anaerobic conditions, especially during exercise. Lactate was previously regarded as “a metabolic waste product” but is now known to be an important fuel source, either used within cells or exported to adjacent organs. Lactate analysis was performed by intra- and extra-cellular analysis and as shown in Fig. 5D, the extra-cellular level was more abundant at 8 h than 24 h with respect to control (p < 0.05); at 8 h lactate level was about 2% respect 24 h. These data suggest that under physiological conditions, MKVC induces a lower intracellular accumulation of lactate with consequent greater consumption of glucose supporting the aerobic activity.

### 3.5 Analysis of the intracellular pathways activated by MKVC on C2C12 cells

**Fig. 6:**
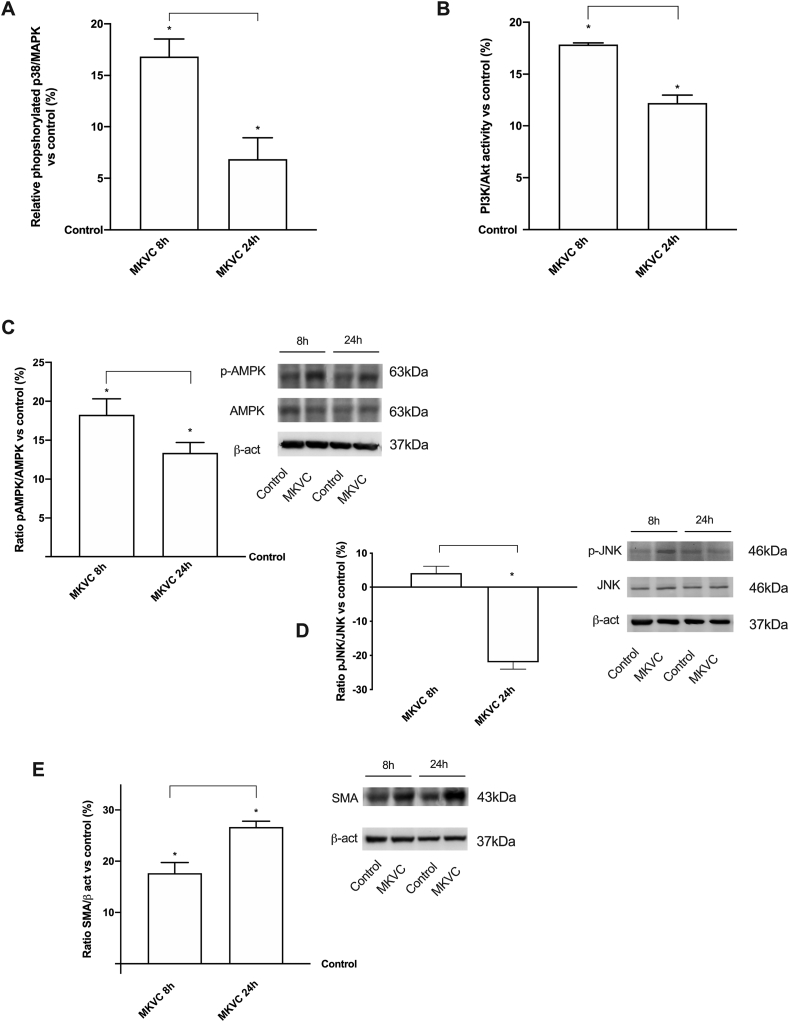
Protein activity, Western blot and densitometric analysis on C2C12 cells. In **A** p38 phosphorylation and in **B** PI3K/Akt activity measured both by ELISA test. Data are reported as means ± SD of four independent experiments reported as % vs control (0 line). The abbreviations are the same as reported in Fig. 1. ∗p < 0.05 vs control; the bar between 8 h and 24 h of MKVC stimulation. In **C** AMPK phosphorylation, in **D** JNK phosphorylation and in **E** SMA expression was analyzed by Western blot and densitometric analysis. The images reported are an example of each protein from five independent experiments normalized on specific total protein when possible and verified by β-actin detection. Data are reported as means ± SD (%) vs control (0 line).

To explore whether MKVC can support different phases and moments in the intracellular mechanisms involved in an exercise, further experiments were performed by analyzing some important kinases in muscle cells. As reported in Fig. 6A, the activity assay of p38/MAPK was analyzed as a marker of contractile response at 8 h and 24 h of stimulations. Data confirmed MKVC's role in aiding the activation of this intracellular pathway at 8 h. Moreover, the effect at 24 h is reduced leading to the energy conservation state. In contrast, the effect at 8 h supported previous findings on the proliferation phase induced by MKVC compared to control (p < 0.05). A second major pathway supporting protein synthesis and glucose uptake, PI3K/Akt (Fig. 6B), confirmed its ability to support a late phase of cell activation since MKVC can induce its activation (p < 0.05) compared to control. Furthermore, at 24 h its activation was reduced compared to 8 h (p < 0.05, about 46%) but it was increased compared to control (p < 0.05, about 12%) indicating that the effects on myoblasts were maintained over time.

Another key regulator of cellular metabolism, which plays a predominant role in catabolic mechanisms is the glucose transporter AMPK, which has also been studied. As reported in Fig. 6C MKVC appears to improve muscle metabolism at 8 h in which a greater expression of AMPK is observed (about 36% than 24 h, p < 0.05) compared to control (p < 0.05, about 18%). The effectiveness of MKVC is maintained over time and demonstrated for the first time that MKVC can induce glucose uptake according to the classic activation mechanisms with mitochondrial modulation. Furthermore, to support the effectiveness of the contraction and to rule out the inflammatory response, the evaluation of JNK ½ was carried out. As reported in Fig. 6D, MKVC improved the biology linked to contractile response, offering a physiological picture of the muscular cell activity at 8 h, it maintained an effect equal to the control which was drastically reduced at 24 h supporting a lower level of hypercontraction with a reduction of 5.5 times in the presence of high glucose. These data support the effectiveness of MKVC in addition to improving metabolism also in reducing any associated inflammatory processes.

Finally, other experiments were conducted evaluating SMA as a marker of muscle regeneration, including post-trauma and anti-muscle damages. As demonstrated in Fig. 6E, after MKVC stimulation, myoblasts appear better at maintaining physiological contractile activities at 8 h as they had a greater effect compared to control (p < 0.05 about 17.7%), and this effect was amplified at 24 h compared to control (p < 0.05) and to 8 h (p < 0.05 about 50.8%), indicating that this effect is consolidated over time. These data support the *in vitro* efficacy of MKVC in maintaining muscle survival systems, even after hypercontraction-related damage.

### 3.6 Simulated hypercontraction induced by caffeine

**Fig. 7:**
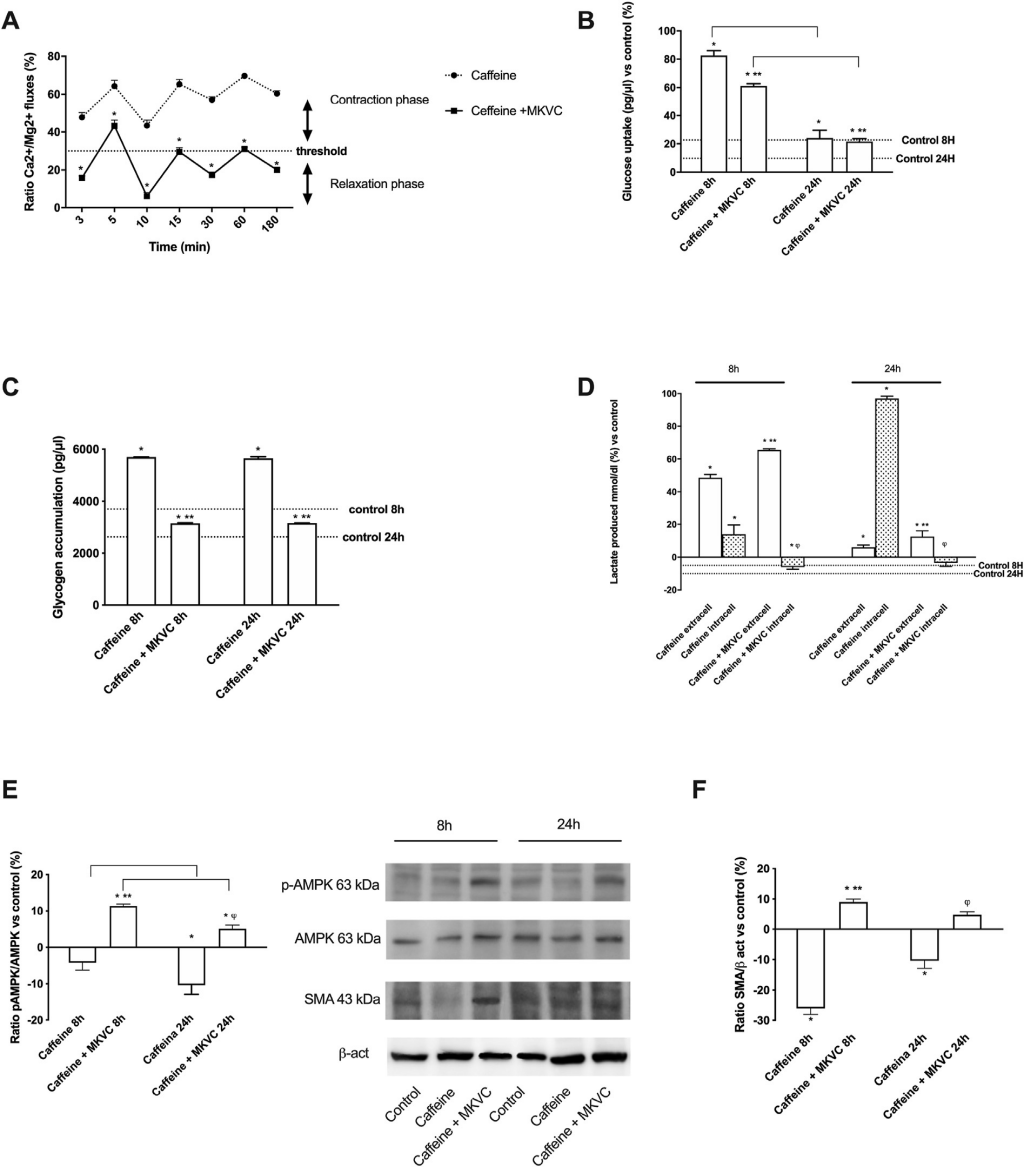
Effects of caffeine on C2C12 cells. In **A** The graph shows a ratio from calcium movements and magnesium flux normalized to control values ranging from 3min to 180min. After the administration (time 0), a series of oscillations of the Ca2 + /Mg2 + ratio was observed, which correspond to cycles of contraction and relaxation of the cells. Data are reported as means ± SD (%) of five independent experiments. In **B** glucose uptake, in **C** glycogen accumulation, and in **D** lactate analysis with intracellular and extracellular quantification. All these parameters were investigated at two time points (8 h and 24 h). Data are reported as means ± SD of five independent experiments. ∗p < 0.05 vs control; ∗∗p < 0.05 vs caffeine; ^φ^ p < 0.05 vs intracellular caffeine; the bars p < 0.05 the same treatment between the different time points. In **E** AMPK phosphorylation and in **F** SMA expression was analyzed by Western blot and densitometric analysis. The images reported are an example of each protein from five independent experiments normalized on specific total protein when possible and verified by β-actin detection. Data are reported as means ± SD (%) vs control (0 line). ∗p < 0.05 vs control; ∗∗p < 0.05 vs caffeine 8 h; ^φ^ p < 0.05 vs caffeine 24 h.

It is well established from *in vitro* studies that caffeine has a direct effect on muscle contraction. It plays a key role in the functioning of the sarcoplasmic reticulum, increasing calcium permeability and making it readily available for the contraction mechanism.^26 33^ Caffeine was administered to the C2C12 cells to provide a ‘strenuous exercise-like treatment’, thus studying the biological aspects of exercise to examine exercise-regulated changes in signal transduction and metabolism. Cells were pre-treated with 2.5 mM caffeine and then treated with MKVC at 8 and 24 h. To understand the effects on contraction-related molecular mechanisms, calcium and magnesium levels were analyzed. As reported in Fig. 7A, caffeine does not allow an adequate relaxation phase. On the other hand, MKVC has a better contraction modulation effect (about 15% less than caffeine), supporting the effects of MKVC in facilitating relaxation after hypercontraction, compared to control (p < 0.05) and compared to caffeine treatment, which instead delays relaxation (p < 0.05). Ultimately, these data state that MKVC better modulates calcium movements, bringing them back to more physiological values and remodeling the contractile phase with less cellular fatigue.

Since *in vivo* exercise induces a cascade of intracellular mechanism (e.g., activation of AMPK), proteomic (e.g., GLUT4), and metabolic changes (e.g., increased glucose uptake), additional experiments *in vitro* were carried out to confirm the effects of MKVC. Consequently, additional parameters such as glucose uptake, glycogen consumption, and lactate accumulation were studied following pre-treatment with caffeine. As shown in Fig. 7B–D, caffeine alone induced a biological response compatible with a state of severe contraction accompanied by high glucose uptake, high glycogen concentration, and a significant increase in extracellular lactate (p < 0.05). This indicates a negative condition of the cells compared to a state of hypercontractility. The stimulation with MKVC was able to reduce this negative condition, leading the extracellular lactate to the control value (p < 0.05 vs caffeine alone).

Since the strong contraction can cause muscle hypertrophy accompanied by cell death in which the stress condition is maintaining for a long time, additional experiments were performed analyzing some intracellular pathways leading to glucose transport (e.g., AMPK) and muscle regeneration (e.g., SMA). As reported in Fig. 7E, following stimulation with caffeine, MKVC allows the restoration of normal glucose uptake with greater effectiveness at 8 h, compared to control (p < 0.05). MKVC was able to modulate the AMPK pathway after caffeine stimulation suggesting its ability to restore and recover muscle cell metabolism after hypercontractility. Finally, the analysis of regeneration and myogenesis markers such as smooth muscle actin (SMA) showed similar results (Fig. 7F). Indeed, MKVC was able to counteract the negative effect of caffeine and the main effects were observed at 8 h compared to control (p < 0.05) and compared to caffeine about twice. In addition, this effect is maintained over time suggesting a long-term effect on the restoration of functionality and on complete post-trauma regeneration.

All these results support the hypothesis that MKVC may play different roles during different phases of muscle activity and reveals beneficial effects during a strong contraction in an *in vitro* model.

## 4 Discussion

Multiple lines of research explored topics directly related to nutrients that further refined information on evidence-based nutrition recommendations to support physical activity.^48^ Use of dietary supplements is widespread in many populations, including athletes, the elderly, sedentary people, and people with chronic diseases often on an empirical basis and without evidence based on experimental data.^49^^,^^50^ For this reason, there is growing interest in research of nutrients/nutraceuticals and associated mechanisms of action concerning skeletal muscle health with the aim of defining evidence-based recommendations. The supplements commonly used for the maintenance of skeletal muscle health are usually made up of proteins and amino acids (e.g., leucine, creatinine, and carnitine), associated with vitamins (e.g., vitamin D or vitamin C) or minerals (e.g., magnesium. or potassium), essential for many metabolic processes.^48^ Recently, however, the need has arisen to develop new types of supplements capable of counteracting muscle damage related to hypercontractility during exercise. For this reason, in this work, the effectiveness of a combination of four substances, magnesium, potassium citrate, vitamin D3, and curcumin (MKVC) has been tested on the biological aspects related to a state of hyper contraction during physical exercise and which can lead to muscle damage. The results of this study show that the combination of these substances exerts a positive effect on myoblasts through the activation of intracellular mechanisms capable of stabilizing the beneficial effects of the individual agents on mitochondrial health. In particular, this effect was observed by the cell viability analysis which can rule out any cytotoxic effect or mitochondrial imbalance in the absence of limited respiration, which otherwise leads to a concomitant high rate of cell apoptosis. In addition, our results support the hypothesis that MKVC can promote physiological differentiation on C2C12 cells as demonstrated by the analysis of cyclin D1, which is abundant in proliferating myoblasts, and confirmed by the analysis of desmin. Furthermore, MKVC shows a significant impact on mitochondrial activity and on its membrane potential because it is capable of increasing ATP production and oxygen consumption. This is a crucial point for the proliferation of myoblasts. Some foods naturally contain a class of nutrients that have beneficial effects on health. These are called nutraceuticals. Skeletal muscle can also benefit from the combination of micro-, macronutrients, and nutraceutical substances both in terms of normal physiological trophism and in terms of response to physical exercise. Consequently, some studies have investigated the potential cooperative effect of combined nutritional “cocktails”.^51^ One of the main areas of interest driving research towards the development of new supplements for the muscle is their possible role in controlling muscle cramps, typical sudden, involuntary, painful, and palpable muscle contractions that last from seconds to minutes. Muscle cramps can be associated with pathological conditions, but most commonly occur in the absence of known conditions.^51^^,^^52^ For this reason, some combinations of micronutrients, such as vitamins and minerals, are being studied to try to reverse this condition. This study fits into this context, in which the importance of the mechanism underlying the muscle contraction-relaxation cycle was further investigated. The time-course study performed on C2C12 cells to analyze Ca2+ and Mg2+ fluxes confirmed the importance of this mechanism in the genesis of cramps, indicating that MKVC better modulates a state of hypercontraction, restoring ion fluxes to more physiological values and remodeling the contractile phase with less cell fatigue in an *in vitro* model. Another important element in the balance between contraction and relaxation is the maintenance of a physiological value of inflammatory cytokines. MKVC can maintain the level of TNFα indicating that it could have a great effect in hypercontractility conditions. Furthermore, the results of this work showed that MKVC induces less intracellular accumulation of lactate, resulting in increased glucose consumption to support aerobic activity in C2C12 cells. These data suggest that glucose concentration and glycogen accumulation are consumed under physiological conditions after MKVC stimulation. Consequently, activation of PI3K/AKT and p38/MAPK confirmed our hypothesis, revealing the role of MKVC in assisting the energy conservation process in C2C12 cells. In particular, the mechanism of action of AMPK demonstrated the efficacy of MKVC in promoting glucose uptake, according to the classic activation mechanisms, which include mitochondrial modulation. Furthermore, the evaluation of JNK ½ and SMA showed that MKVC supports better maintenance of muscle cell survival systems, assuming greater efficiency even after contractile damage. The stimulation with caffeine was aimed at mimicking a state of hyper contraction which during exercise can lead to the onset of muscle cramps. Caffeine is a potent metabolic stimulant in skeletal muscle that has ergogenic effects and has been shown to stimulate several exercise-like effects within skeletal muscle cells.^53^ *In vitro* skeletal muscle treatment with caffeine promotes insulin-independent glucose transport, fatty acid oxidation, the release of Ca2+ from the SR, and mitochondrial biogenesis which is the basis of the contraction skeletal muscle*.* Caffeine treatment has been applied to skeletal muscle cells that have Ca2+ ionophore, with the specific intent of obtaining an exercise-like treatment, mimicking exercise activation and trigger Ca2+ changes like those found in exercise, mimicking the typical activation of exercise or exercise signals and trigger Ca2 + changes similar to those in exercise. For this reason, caffeine has been a widely accepted experimental model for examining exercise-regulating changes in signal transduction and metabolism in skeletal muscle cells.^54^ Our results revealed that MKVC is able to counteract the negative effect of caffeine, suggesting a long-term effect on muscle function restoration and complete post-trauma regeneration in an *in vitro* model. MKVC treatment, with or without caffeine pre-stimulation, supports the hypothesis that a new supplement based on the MKCV combination may have numerous roles during cell muscle activity and reveals beneficial effects on the biology of the muscle cells in the strong state contraction. Since some criticisms have been raised for the stimulation of caffein additional experiments will be necessary in order confirm the present findings on *in vivo* model, which may be a pre-clinical option that precede MKVC human use.

## 5 Conclusion

In conclusion, all these findings reveal for the first time that the combination of magnesium, potassium citrate, vitamin D3, and curcumin exert beneficial effects on skeletal muscle cells under physiological conditions and during strenuous activity, indicating that it may be the good choice for muscle exercise support.

Further study *in vivo* model may be useful to establish whether this protective mechanism activated by MKVC can also bring beneficial effects in the physiological loss of muscle mass, called sarcopenia, which occurs with advancing age.

## Funding

This project is supported by private funding of lab Physiology.

## Declaration of competing interest

The authors declare no conflict of interest.

## Declaration of competing interest

All the authors declare no conflict of interest.
