# Supplementary material for: Preventing c2c12 muscular cells damage combining magnesium and potassium with vitamin D3 and curcumin
Source: J Tradit Complement Med. 2021 Jun 2;11(6):532–44. doi: 10.1016/j.jtcme.2021.05.003 (PMC8572722; doi:10.1016/j.jtcme.2021.05.003)
Supplement: Multimedia component 1 [file mmc1.docx]

**Supplementary Material**

**Supplementary 1. Cell viability**

The cells were incubated in DMEM without red phenol and 0% FBS with 1% MTT dye for 2h at 37 °C with 5% CO2. Then, the formazan crystals were solubilized by Solubilization Solution; the cell viability was determined by measuring the absorbance through a spectrometer (VICTORX4 multilabel plate reader) at 570 nm with correction at 690 nm. Cell viability was obtained by comparing results to control cells and expressed as a percentage (100% viable).

**Supplementary 2. Oxygen Consumption and Mitochondrial Membrane Potential**

Oxygen consumption fluorescence was measured by excitation/emission at 380/650 nm, and the membrane potential was measured using JC-1 red aggregates at an excitation/emission of 650/690 nm and green monomers at an excitation/emission of 485/535 nm in a fluorescence spectrometer (VICTORX4 multilabel plate reader). The results about Jc-1 are expressed as red fluorescence means ± SD and the results about oxygen consumption are expressed as means ± SD (%) compared to control cells.^40^

Supplementary 3. ATP assay

At the end of each stimulation, the cells were immediately treated with the components of the ATP assay kit (Calbiochem, San Diego, USA). Luminescence was measured in one minute after the addition of ATP monitoring enzyme in a VICTORX4 multilabel plate reader, and luminescence was calculated as μmol of ATP/g protein^40^ and reported as means ± SD of nanomol (nmol) per well.

**Supplementary 4. TNFα assay**

100 μL of samples were added to each well of a 96-well ELISA plate. The plate was incubated at room temperature for 2h followed by overnight incubation at 4°C. The wells were washed, incubated with 100 μL of biotinylated anti-TNFα for 2h incubation at room temperature, washed and incubated with 100 μL Streptavidin-HRP for 1h. After that, the plate was washed and incubated with 100 μL of chromogen solution for 30 minutes at room temperature in the dark. The absorbance was measured after the addition of stop solution at 450 nm using a plate reader (VICTORX4 multilabel plate reader).^42^

Supplementary 5. Glucose uptake

Cells were lysed in the extraction buffer by several freeze/thaw cycles and then heat at 85 °C for 40 min. The cooling cell lysate was put on ice for 5 minutes, neutralized by adding 10 μL of Neutralization Buffer, spun at 13,000 × g to remove insoluble material, then the amount of glucose in the lysate was measured reacting with mixed reagent A and B (Glucose Uptake Colorimetric Assay Kit, Sigma-Aldrich). The absorbance was measured at 412 nm by spectrophotometer (VICTORX4 multilabel plate reader) and data reported as means±SD of pmol/µl derived by standard curve.^43^

Supplementary 6. Glycogen measurement

C2C12 cells were lysed and boiled for 5 min to inactivate enzymes as reported in the literature (BioVision, Life Research, Scoresby Victoria, Australia).^37^ Samples were centrifuged at 13000 rpm for 5 min and the supernatant was pick up to induce the hydrolysis of glycogen to glucose in presence of OxiRed probe to generate color (lmax = 570 nm). The glycogen concentration in the samples was calculated by C = Ay/Sv where Ay is the amount of glycogen (mg) in the sample as determined from a standard curve and Sv is the sample volume (ml).^44^

Supplementary 7. Akt Activation Assay

50 μL/well of each lysed sample was added in InstantOne ELISA microplate strips for 1 h at room temperature on a microplate shaker with the antibody cocktail. At the end, the detection reagent was added for 20 min and then stopped by adding a stop solution. The strips were measured by a spectrometer (VICTOR X4 multilabel plate reader) at 450 nm. The results were reported as mean absorbance (%) compared to control.

Supplementary 8. Phospho-p38/MAPK ELISA test

At the end of stimulations, cells were lysed to evaluate phosphorylation levels of p38/MAPK using a cold Lysis Buffer kit supplemented with protease and phosphatase inhibitors, following the manufacturer’s instructions (p38 MAPK alpha (pT180/pY182) + total p38 MAPK alpha ELISA Kit, Abcam, ab221012).^47^ Absorbance was read at 450 nm on a microplate reader and the results were reported as means (%) vs control.
